# Evaluating the behavioural consequences of external pop‐off biologging packages on two freshwater fishes

**DOI:** 10.1111/jfb.70560

**Published:** 2026-07-07

**Authors:** L. LaRochelle, G. D. Raby, J. W. Brownscombe, M. B. Shorgan, A. T. Fisk, A. J. Danylchuk, S. J. Cooke

**Affiliations:** ^1^ Fish Ecology and Conservation Physiology Laboratory, Department of Biology and Institute of Environmental and Interdisciplinary Science Carleton University Ottawa Ontario Canada; ^2^ Integrative Fish Ecology Laboratory, Department of Biology Trent University Peterborough Ontario Canada; ^3^ Great Lakes Laboratory for Fisheries and Aquatic Sciences Fisheries and Oceans Canada Burlington Ontario Canada; ^4^ School of the Environment University of Windsor Windsor Ontario Canada; ^5^ Department of Environmental Conservation University of Massachusetts Amherst Amherst Massachusetts USA

**Keywords:** acoustic telemetry, biologging, fishing, post‐release behaviour, tagging effect

## Abstract

Quantifying the behaviour of animals in the wild can provide important information on their ecology and welfare. External biologgers that ‘pop‐off’ to enable tag retrieval have been widely used in marine systems; however, they have only recently been applied to freshwater fishes. As a result, questions remain about the impact that external biologgers have on the behaviour of freshwater fishes. Here, two species of freshwater fish that were previously implanted with acoustic transmitters were recaptured with rod‐and‐reel and fitted with custom pop‐off biologging packages (50 × 30 × 15 mm; 14.7 g) in a small (23 ha) lake. Habitat use and movement behaviour (e.g., cruising, exploratory, stationary) were assessed for each individual for 72 h when pop‐off biologging packages were affixed to the fish, and once they were detached from the fish. Acoustically tagged fish in the system that were never equipped with a pop‐off biologging package were included as controls. Behavioural impacts of pop‐off biologging packages were more evident for Largemouth Bass *Micropterus nigricans* than for Northern Pike *Esox lucius* in water temperatures between 20 and 27°C. Largemouth Bass spent less time in the limnetic habitat and more time close to refuge when pop‐off biologging packages were attached compared to controls or when they were detached. Northern Pike spent more time in the limnetic habitat and less time in the semi‐complex habitat when pop‐off biologging packages were attached compared to controls. Pop‐off biologging packages had no impact on the movement behaviour of Northern Pike. Largemouth Bass attached with pop‐off biologging packages were stationary for longer compared to controls, or once the pop‐off biologging packages were detached. Once the pop‐off biologging packages detached, Largemouth Bass movement behaviour resembled the controls, which spent more time cruising and exploring. These results provide important information on behavioural artefacts that can manifest when wild fish are equipped with short‐term external biologging packages.

## 1 INTRODUCTION

External pop‐off biologging packages or similar style packages (e.g., satellite tags) are commonly used in marine systems (Lear & Whitney, 2016; Robichaud et al., 2025; Whitney et al., 2016), yet external pop‐off packages are rarely used for freshwater fish (LaRochelle et al., 2025). There are two factors that make the use of external pop‐off packages in freshwater challenging: the size of the animal relative to the size of the external tagging packages and a lack of reliable release mechanisms. In marine systems, there are more species that reach large body sizes and can thus accommodate larger tags. Smaller fish are unable to accommodate large external packages since hydrodynamic drag may impact energy expenditure (Lynch et al., 2017; Methling et al., 2011; Tudorache et al., 2014) and swimming performance (Janak et al., 2012; McCleave & Stred, 1975; Peake et al., 1997). The amount of drag caused by external tags is influenced by the shape (e.g., Shorter et al., 2014), size (e.g., Zhang et al., 2020) and location of the tag on the body (e.g., Tudorache et al., 2014). Additionally, time‐released galvanic clips that rely on corrosion work poorly (or not at all) in freshwater, making it difficult for the pop‐off packages to disconnect from fish and float to the surface for recovery. It is becoming increasingly feasible to use pop‐off biologging packages on freshwater fishes as technological advances have allowed for the miniaturization of biologging devices (e.g., Elliot et al., 2023; Raby et al., 2017) and the development of creative release mechanisms (e.g., circular candy; LaRochelle et al., 2024, catgut suture; LaRochelle et al., 2023; Wilson et al., 2026).

Although external pop‐off biologging techniques have been used to assess the behaviour of freshwater fishes (e.g., Elliot et al., 2023; LaRochelle et al., 2023; LaRochelle et al., 2024; Raby et al., 2017), the effects that external biologgers have on the behaviour of freshwater fishes are unknown (Jepsen et al., 2015; LaRochelle et al., 2025). Indeed, there is mixed evidence of potential behavioural impacts and energetic costs associated with external tag attachment in fishes. Small external pop‐off satellite tags did not have adverse effects on the migration of European Sea Bass *Dicentrachus labrax* (O'Neill et al., 2018) and Atlantic Salmon *Salmo salar* (Hedger et al., 2017) but did reduce the diving ability of Atlantic Salmon at sea (Hedger et al., 2017). External tags led to a 26% increase in energy use during swimming for European Eels *Anguilla anguilla* (Methling et al., 2011). Echave (2016) observed Sablefish *Anoplopoma fimbra* in a laboratory setting following the attachment of a pop‐off satellite package and found no impacts on the normal behaviour of those fish (e.g., feeding, or physical damages) associated with attachment. A general caveat to most tagging effects studies is that they focus on one endpoint (e.g., swimming performance) and often miss potential behavioural implications related to tagging (Shorgan et al., 2026). Understanding the influences that external biologging packages have on the behaviour of freshwater fishes in the wild will provide important considerations for behaviour interpretations in future studies.

Assessing behavioural traits in fish in the wild can provide valuable insight into their welfare (Beitinger, 1990; Huntingford et al., 2006; Iwama et al., 1997). There are fitness and survival implications depending on the magnitude of behavioural deviation in wild fish (Schreck et al., 1997). Behavioural differences can be observed when swimming capabilities are reduced (Thompson et al., 2008) or when fish engage in hyperactivity (Mäkinen et al., 2000; LaRochelle et al., 2025). Cognitive impairment and disorientation can result in greater susceptibility to predation as abilities to find refuge and avoid predators is reduced (Cooke et al., 2014; Danylchuk et al., 2007). Exploration is energetically costly and potentially increases susceptibility to predation (Mikheev et al., 1997; Mikheev et al., 2010). However, fish must move and explore within their environment to fulfil fundamental living requirements (e.g., food acquisition). Feeding efficacy has previously been reported to increase when searching for food in open limnetic habitats (Mikheev et al., 2010). When faced with predation risk, fish tend to use habitats that are more complex and provide refuge (Eklöv, 1997; Savino & Stein, 1982; reviewed in Mikheev et al., 2010). Alternatively, when fish are willing to engage in more bold behaviours and make themselves risk‐prone, they will explore limnetic habitats where refuge offered by complex habitats is not available (Budaev, 1997). This bold behaviour while exploring the limnetic zone is adaptive and can change depending on the situation (Wilson, 1998). Activity levels are generally elevated when fish are in open limnetic zones (Enefalk & Bergman, 2016; Radabaugh et al., 2010) compared to when in complex structures where movement is obstructed, increasing the energetic cost of movement (Brownsmith, 1977; Schooley et al., 1996). Further, stress levels can impact the physiological abilities and cognitive actions of fishes (reviewed in Cooke et al., 2022). Therefore, movement of fish within an aquatic system is a balance between energy mobilization, or the cost of movement and the risk/benefits of moving. Identifying patterns of habitat use and movement behaviour of fish with and without external pop‐off biologging packages can provide critical insight into fitness implications when fish are equipped with external pop‐off biologging packages.

Biologgers, also known as data storage tags or archival tags, are able to record high‐resolution/high‐frequency data. There are several different ways that biologgers can be attached or inserted into fish, and they all have varying strengths and weaknesses. For example, biologgers can be placed in the body cavity via surgery (Clark et al., 2010; Reeve et al., 2025), attached on the outside of the fish with a detachable strap (LaRochelle et al., 2021; Louison et al., 2023; Madden et al., 2025; Reid et al., 2025), attached through the dorsal musculature (Algera et al., 2017; Boudreau et al., 2025) or with a pop‐off package mechanism (Brewster et al., 2021; Danylchuk et al., 2026; LaRochelle et al., 2023). These tags include one or more sensors and are typically multi‐channel such that data from different sensors can be recorded simultaneously (Williams et al., 2020). However, because they are archival tags, it is imperative that the biologgers are recovered if those data are to be collected. On the other hand, acoustic telemetry is a passive technology that records the detection of fish across an acoustic array of hydrophones. Generally, acoustic transmitters are inserted into the body cavity of the fish, requiring surgery and subsequent recovery, yielding a high retention rate (Shorgan et al., 2026). Because data is transmitted to stationary receivers, the transmitters do not need to be recaptured or recovered to obtain data (Hussey et al., 2015). However, the acoustic array must cover the majority of the area occupied by the fish, or else there will be no data collected. Both technologies have strengths and weaknesses, which need to be considered when designing a study (reviewed in Cooke et al., 2012).

The objective of this study was to determine if custom external pop‐off biologging packages (50 × 30 × 15 mm; 14.7 g) alter the behaviour of Largemouth Bass *Micropterus nigricans* and Northern Pike *Esox lucius* in the wild. These two species were selected given that previous studies have equipped them with pop‐off biologging packages (e.g., LaRochelle et al., 2023; LaRochelle et al., 2024; LaRochelle et al., 2026; Wilson et al., 2026). Here, we used Largemouth Bass and Northern Pike that were previously tagged with internal acoustic transmitters and equipped them with external pop‐off biologging packages. Using fish that were recaptured and recovered from the surgical tagging process provided a unique way to isolate and assess the impacts that external pop‐off biologging packages had on the behaviour of fish in the wild. We used high‐resolution positions (every 14–18 s) from the acoustic telemetry array to assess habitat use and different movement behaviours (e.g., tortuosity, step length distance and linear displacement). Overall, these findings will provide valuable information on the baseline behaviour of fish equipped with external pop‐off biologging packages that should be considered when contextualizing the behaviour of fish in the wild when similar biologging techniques are used.

## 2 METHODS

### 2.1 Acoustic tagging

**FIGURE 1:**
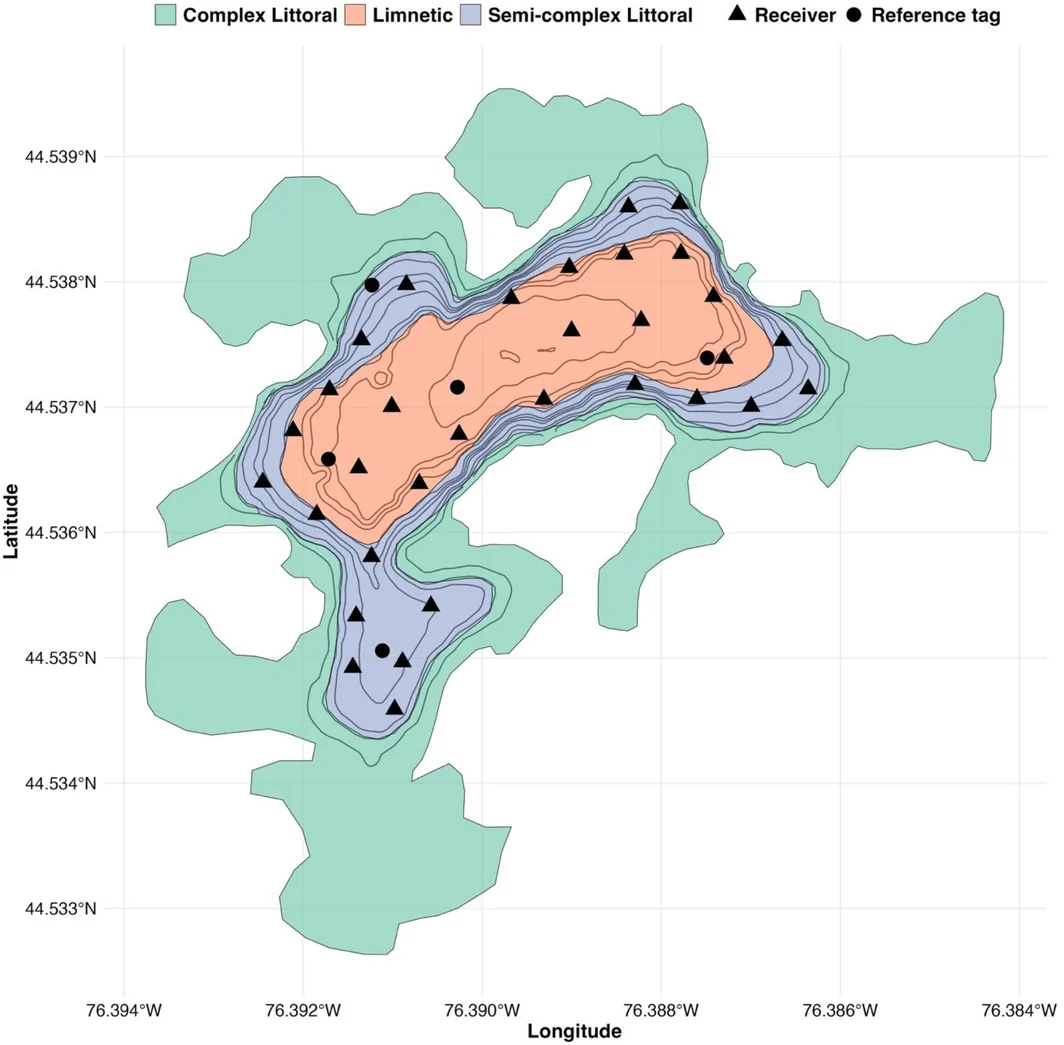
Habitat and bathymetry map (2 m contour lines) of Lindsay Lake. The different colours represent the various habitat types in the lake. Black triangles (*n* = 32) indicate the receiver locations, and black points indicate the location of reference tags (*n* = 5).

Tagging practices were approved by Carleton University (AUP #110723) and in accordance with the Canadian Council of Animal Care. Lindsay Lake, a small research lake (23 ha) located at Queen's University Biological Station (44°32′14.0″ N, 76°23′23.1″ W; Figure 1), was equipped with 32 high‐residency acoustic receivers (307 kHz HR3; InnovaSea Systems Inc., Halifax, NS, Canada). The receivers were strategically placed, allowing for triangulation of detections that provided the fine‐scale positioning of fish using Innovasea's High Residency system. Each receiver was positioned approximately 75 m from adjacent receivers and approximately halfway down the water column.

Between 4 and 5 May 2023, Largemouth Bass [*n = 24*, 431 ± 32 mm total length (mean ± SD), 376–495 mm range] and Northern Pike [*n = 19*, 655 ± 59 mm total length (mean ± SD), 545–735 mm range] were captured by rod and reel and brought to shore for surgical tag implantation using a submerged recovery bag (Dynamic Aqua Ltd., Surrey, BC; 122 × 31 cm with a mesh of 0.5 cm on both ends). Fish were then transported to a surgery trough lined with a Transcutaneous Electrical Nerve Stimulation unit (TENS Units INC, Largo, Florida, USA) to temporarily immobilize fish during surgery (see Reid et al., 2019). The TENS unit was set to a constant pulse rate of 150 Hz and a pulse width of 300 μs. Electrical intensity was incrementally increased until fish were immobilized at which point surgery was initiated (Reid et al., 2019; Reid et al., 2024). A tube with fresh recirculating water was placed into the mouth of the fish, allowing for oxygenated water to flow over the gills. To inset the tags in the coelomic cavity, a small (<1 cm) incision was made between the pelvic fins and the cloaca. Fish were equipped with V6‐4× 307 kHz acoustic transmitters (low power 145 dB, 14–18 s delay, 290‐day life, 19.3 mm length × 6.3 mm diameter, 1.2 g in air; InnovaSea Systems Inc.), as well as a passive integrated transponder (PIT; 12 mm; APT12 Biomark, Boise, Idaho, USA) for identification. The incision was closed with a 3‐2‐2 surgeon's knot using 3‐0 absorbable monofilament PDS II sutures (Ethicon, New Jersey, USA). Surgeries took less than 2 min, and all tools were sterilized with betadine prior to tagging. Fish were then released and allowed to recover for at least 1 month prior to recapture, well beyond the recovery threshold of ~200 h identified in the same species and system (Shorgan et al., 2025).

### 2.2 Habitat mapping

**FIGURE 2:**
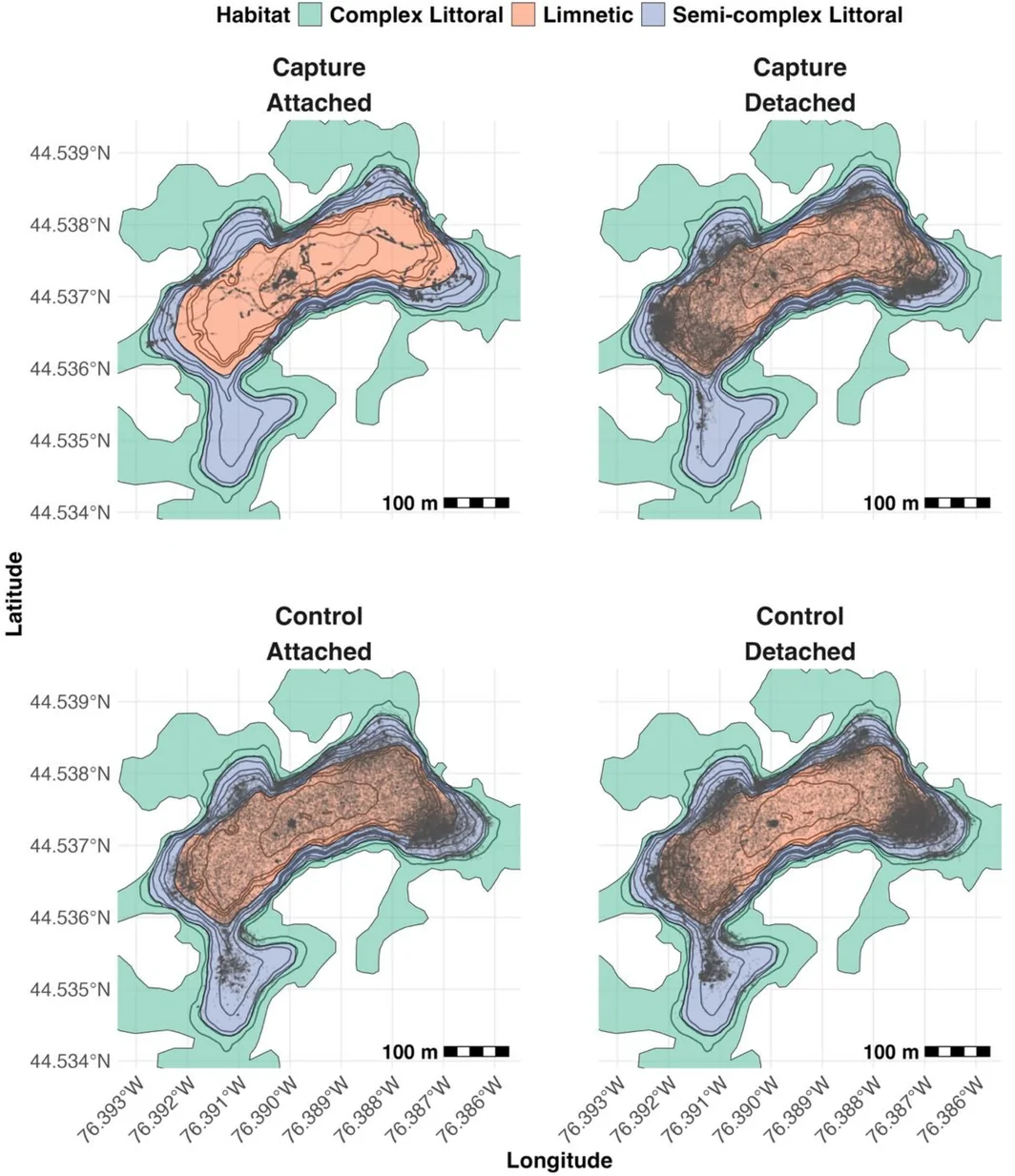
Transparent grey points represent the location of captured (*n =* 6) and control (*n =* 6) Largemouth Bass *Micropterus nigricans* while pop‐off biologging packages were attached or detached during the same periods. The number of detections is weighted the same between the captured and control fish.

**FIGURE 3:**
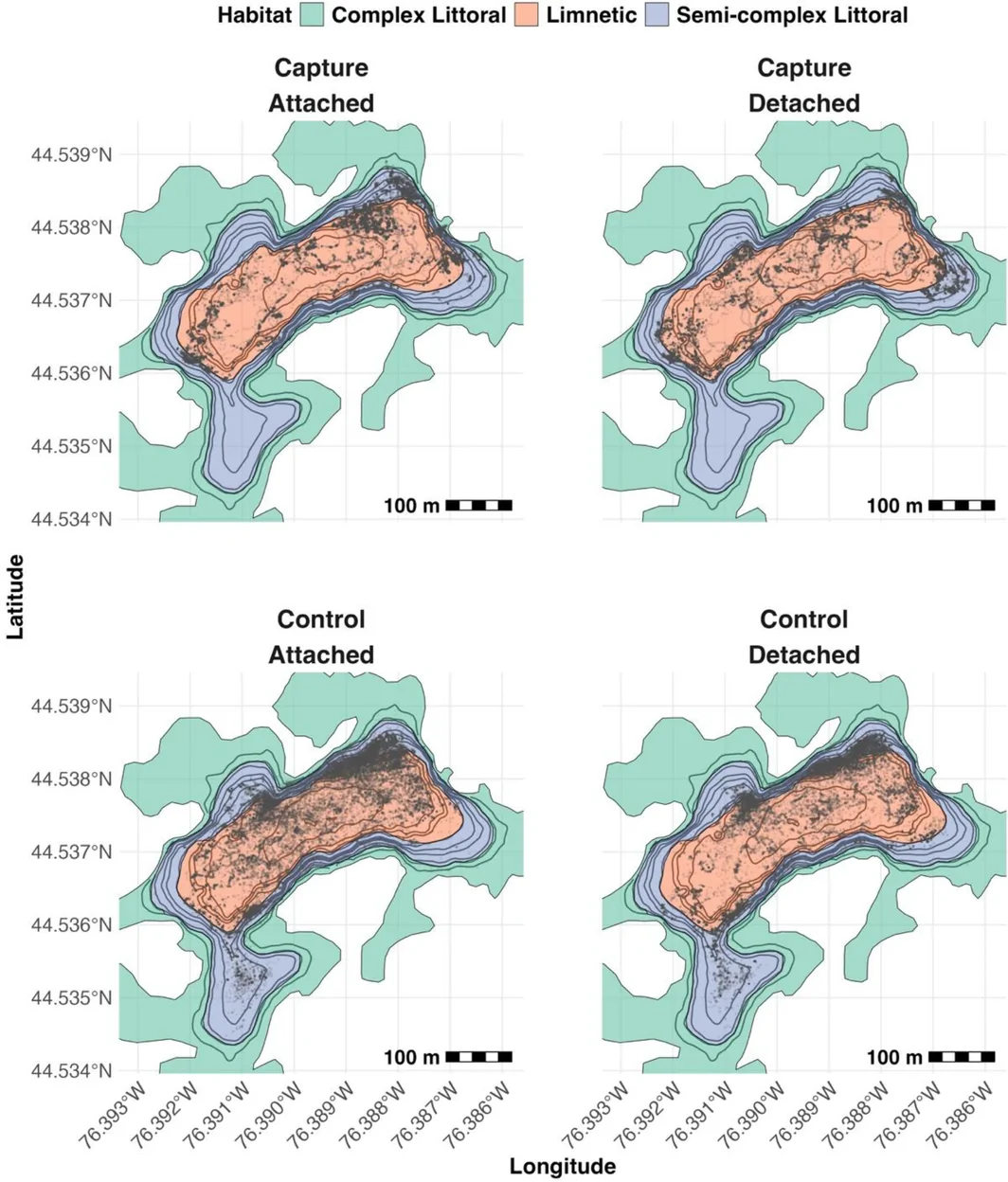
Transparent grey points represent the location of captured (*n =* 6) and control (*n =* 4) Northern Pike *Esox lucius* while pop‐off biologging packages were attached and detached during the same periods. The number of detections is weighted the same between the captured and control fish.

Habitat types within Lindsay Lake were assigned by creating polygons in RStudio using the *editMap* function from the *mapedit* package (Applehans et al., 2026). These polygons were created using the combination of bathymetry and vegetation regime within the lake. Polygons were classified into three categories: complex littoral, semi‐complex littoral and limnetic (Figure 1). Complex littoral zone (13.4 ha) was classified as areas that were heavily vegetated (areas with abundant floating vegetation), woody debris or areas that were <1.5 m. Semi‐complex littoral zone (4.8 ha) was classified as areas with submerged vegetation or between 1.5 m and <4.8 m. Finally, the limnetic zone (5.1 ha) was classified as the area in the middle of the lake that was >4.8 m without any vegetative structure. These habitats provided various levels of refuge, whereby the complex littoral zone had the greatest amount of refuge, followed by the semi‐complex littoral zone; refuge was minimal in the limnetic zone. The acoustic receiver array had an efficient detection range for the limnetic and semi‐complex habitats, but detection efficiency was limited in the complex littoral habitat (Figures 2 and 3).

### 2.3 Recapture and external tagging

Largemouth Bass (*n* = 6; mea*n* = 426 mm ± 39 SD, range = 383–481 mm) and Northern Pike (*n* = 6; 586 mm ± 31, 569–635 mm) were recaptured between 38 and 114 days post‐tagging (11 June and 2 September 2023), using rod and reel. Largemouth Bass (*n = 6*; 450 mm ± 20, 410–460 mm) and Northern Pike (*n* = 4; 713 mm ± 25, 690–735 mm) that were not recaptured and equipped with a pop‐off biologging package were used as controls. Surface water temperature during the angling period varied between 20 and 27°C. Fish were captured on a variety of lures, including bladed jigs, topwater lures and soft plastic lures (rigged with size 1/0 octopus or extra wide gape hooks). All fish were netted and unhooked in <20 s, with total air exposure prior to release being <60 s. All captured fish were then transferred onboard the vessel to a water‐filled V‐trough where they were scanned for their unique PIT tag.

**FIGURE 4:**
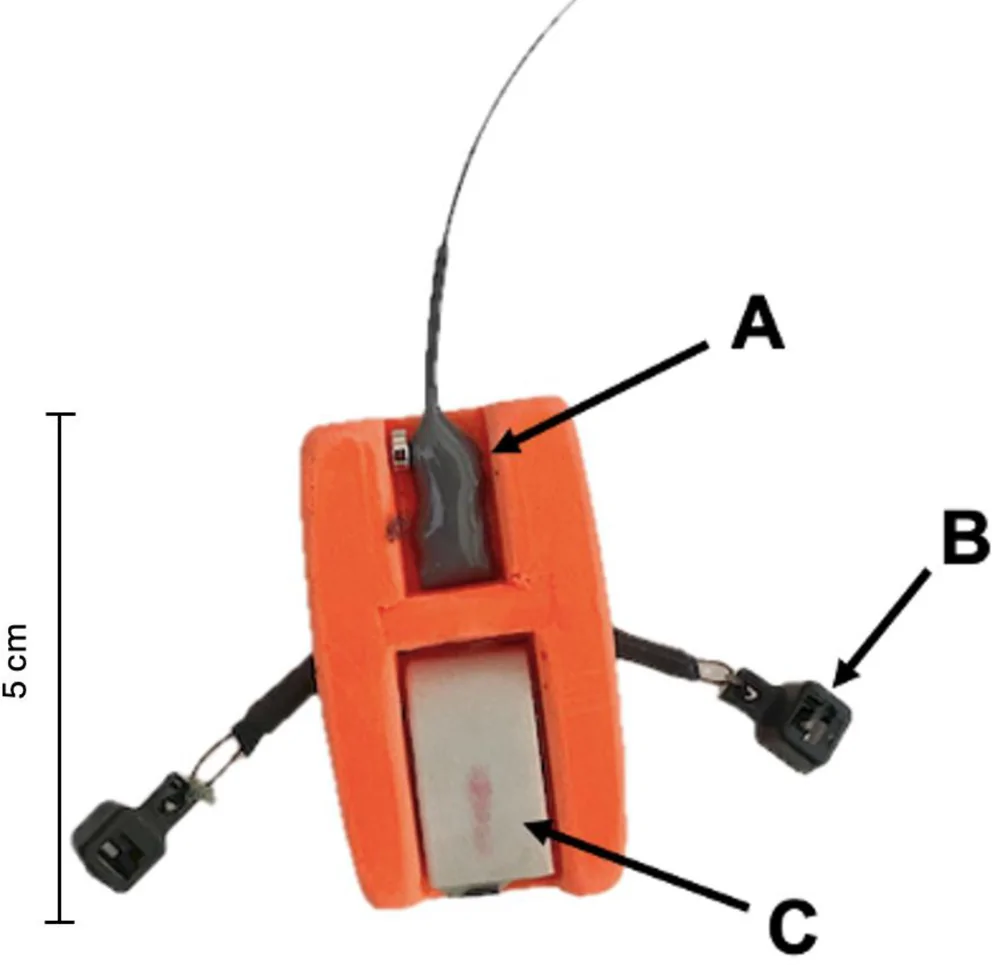
Custom floating pop‐off biologging package used to assess the behaviour of fish in the wild. This package contains a radio transmitter (a), an internal data storage tag (b) and receptacles for self‐locking straps (c) used to affix the biologging package to fish.

**FIGURE 5:**
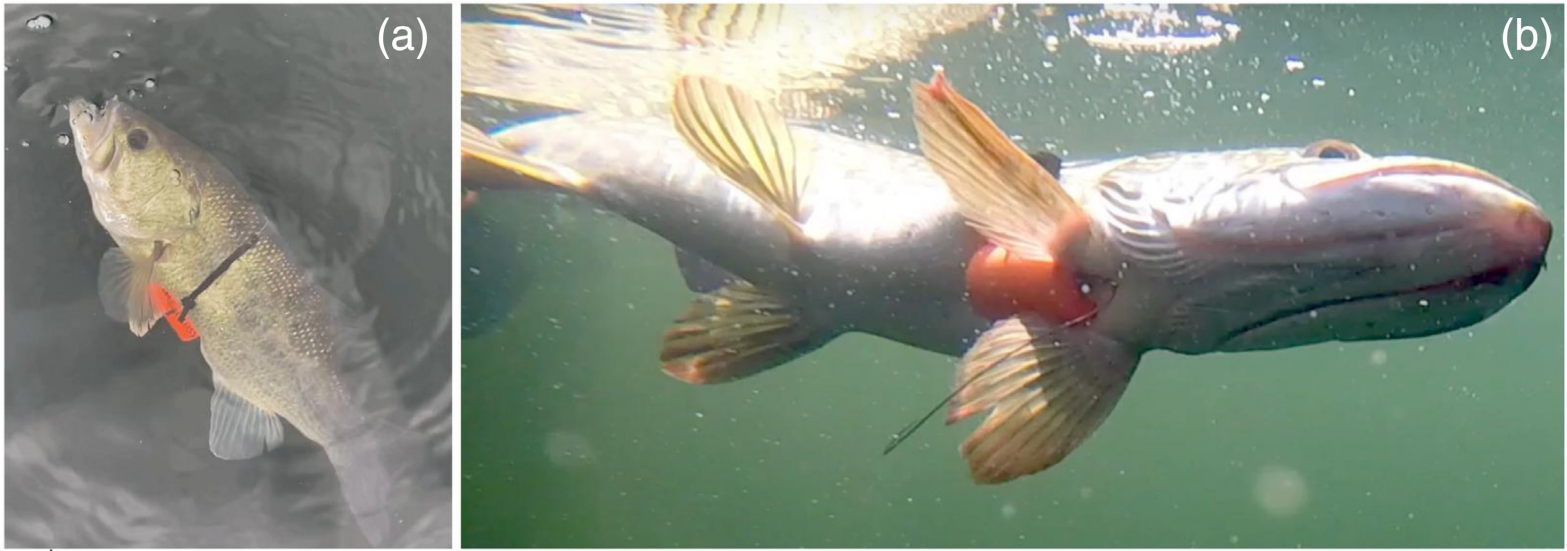
Release photos of a Largemouth Bass *Micropterus nigricans* (a) and a Northern Pike *Esox lucius* (b) with a pop‐off biologging package affixed between their pectoral fins.

Each recaptured fish was equipped with a custom floating pop‐off biologging package (Figure 4; 50 × 30 × 15 mm; 11 g; casing without biologger) made from orange dyed Smooth‐on Feather Lite™ (Smooth‐on, Inc., Macungie, Pennsylvania, USA). The pop‐off biologging package consisted of a data storage tag (AXY‐5 XS; TechnoSmArt, Guidonia Montecelio, Italy; 20 × 10 × 6 mm; 2.5 g in air) and a radio transmitter (BD‐2; Holohil, Carp, Ontario, Canada; 1.2 g in air) used for relocation. These were affixed ventrally between the pelvic fins of Largemouth Bass and pectoral fins of Northern Pike (Figure 5; also see LaRochelle et al., 2023; LaRochelle et al., 2024). Biologging packages were fastened to the fish using two self‐locking straps that were secured together using a short (~3 cm) piece of 4‐0 plain catgut suture (550B, Ethicon, Sommerville, New Jersey, USA), which was used as a dissolvable link. The self‐locking straps were then placed into the matching receptacle that was attached to the pop‐off biologging package and tightened onto the fish. Pop‐off biologging packages would dislodge from the fish when the dissolvable link broke or lost tensile strength, and they would float to the surface for collection. Fish remained submerged during the entire process of affixing the pop‐off biologging packages, which took <45 s. Detachment of biologging packages was identified using depth data from the biologgers. The pop‐off biologging package was deemed to be detached from the fish when there was a steady decrease in depth, and the depth remained at 0 m (i.e., the biologger was at the surface). The last time point prior to the constant decrease in depth was used as the timestamp for being attached to a fish. Pop‐off biologging packages had a relative size of 12% of the mean total length for Largemouth Bass and 8% for the Northern Pike.

### 2.4 Acoustic telemetry monitoring

Fine‐scale positing estimates for both species were created using the Innovasea High Residency system using hyperbolic positions based on the timing of detections (Smith, 2013). Five reference transmitters were used to calculate the estimated horizontal position error (HPE; Figure 1), which was used as an estimated positional error for each fish position (Smith, 2013). HPE was calculated using the horizontal distance between a synthesized position [i.e., measured HPE in m–(HPEm)] of reference tags and the known receiver location. Based on the HPEm from the reference tags, we removed all detections with an HPE >1.5 (Smith, 2013). This HPE cut‐off had a mean HPEm of 1.8 m, with 99% of the measured errors being below 5.8 m. Two‐dimensional positions had a median interval of 16 s.

To avoid confounding impacts from the angling event on the behaviour of fish, only fish that retained their pop‐off biologging packages until after behavioural recovery from angling were used in the analyses (LaRochelle et al., 2026). Based on previous results on behavioural recovery, Largemouth Bass that retained pop‐off biologging packages for 9.5 days and Northern Pike that retained pop‐off biologging packages for 9 days were used. Of the fish used for this study, Largemouth Bass retained their pop‐off biologging packages for a median duration of 245 h (± 67 SD), and Northern Pike retained pop‐off biologging packages for a median duration of 281 h (± 83 SD).

Habitat use by each fish was determined by calculating the total number of minutes each fish spent in the complex littoral, semi‐complex littoral and limnetic zones, or the amount of time not positioned for each hour of the 144‐h monitoring period (72 h with the pop‐off biologging package attached, 72 h after detachment). The total time both fish species were not positioned within the array was also included as an undetected period to account for the period that the fish were not being positioned within the array. Control fish were not separated into the attached and detached periods. Given the high detectability of fish in the middle of the lake (Figures 2 and 3), fish that were not detected were assumed to be in the complex littoral zone. The proportion of time per hour that fish were not positioned was also included in the pop‐off biologging package status variable for modelling. Positions of fish for each hour were only included if they were positioned within 5 min of the previous positioning. Movement was assessed across all hours during the monitoring period by calculating the step length, net displacement and the tortuosity for each individual and hour. Total step length was calculated by summing the distance between consecutive points for each hour, providing the overall movement. The net displacement was calculated as the distance between the initial and final locations of the animal within a given hour. Finally, tortuosity was used to determine how direct or indirect the movement behaviour (i.e., straightness or sinuosity; Bovet & Benhamou, 1988; Benhamou, 2004; Postlethwaite et al., 2013) of the fish was during each hour. For tortuosity, values near 1 suggest straighter swimming paths, while values nearing 0 represent swimming paths that are more tortuous. The tortuosity was calculated as a ratio by dividing the net displacement by the total step length for each hour for each fish.

### 2.5 Statistical analysis

All statistical analyses were performed in R version 4.5.1 (R Core Team, 2025) via R studio (2025.05.1.153; Posit team, 2025). Plots were created using the *ggplot2* package (Wickham, 2016). Pairwise comparisons were used to assess differences between the pop‐off biologging package status (i.e., attached, detached and control) for each model using the *contrast* function from the *emmeans* package (Lenth, 2025). Significance threshold was α = 0.05 for all models and post‐hoc testing.

Proportion of time among habitats was modelled for both species with generalized linear models with a beta regression distribution using the *glmmTMB* function from the *glmmTMB* package (Brooks et al., 2017). For both the Largemouth Bass and Northern Pike, generalized mixed effects models were fit with the interaction of pop‐off biologging package status (i.e., tag attached, detached and control) and habitat type (i.e., undetected, complex littoral, semi‐complex littoral and limnetic) as fixed effects. These models also included fish ID as a random effect to account for repeated measures of individuals.

We then used k‐means clustering to identify and classify different behaviour types based on the tortuosity, step length displacement and net displacement of both species. The same three behaviour types were identified (cruising, exploring and stationary) and used across both species for the analyses. Cruising consisted of long linear displacements with little tortuosity (i.e., straight swimming), exploring was identified when fish had a short net displacement, but a long step length and high tortuosity (i.e., several turns in a localized area), and stationary was identified with minimal net and step length displacement. To assess if the identified behaviour types (e.g., cruising, exploratory and stationary) from the k‐means clustering differed across the status of pop‐off biologging packages (e.g., attached, detached, control), three separate generalized linear mixed models were fit using the *glmer* function from the *lme4* package (Bates et al., 2015). All models were fit with pop‐off biologging package status as the fixed variable, and fish ID as a random variable to account for the repeated measures on individual fish. These models used a binomial distribution because the data consisted of counts for each fish across the hours of the monitoring period. For each fish at each given hour, the behavioural type identified was assigned a 1, while a 0 was assigned to the other behaviour types for that fish in that given hour. This method allowed us to calculate the proportion of time spent engaging in the different behaviour types.

## 3 RESULTS

### 3.1 Largemouth bass

**FIGURE 6:**
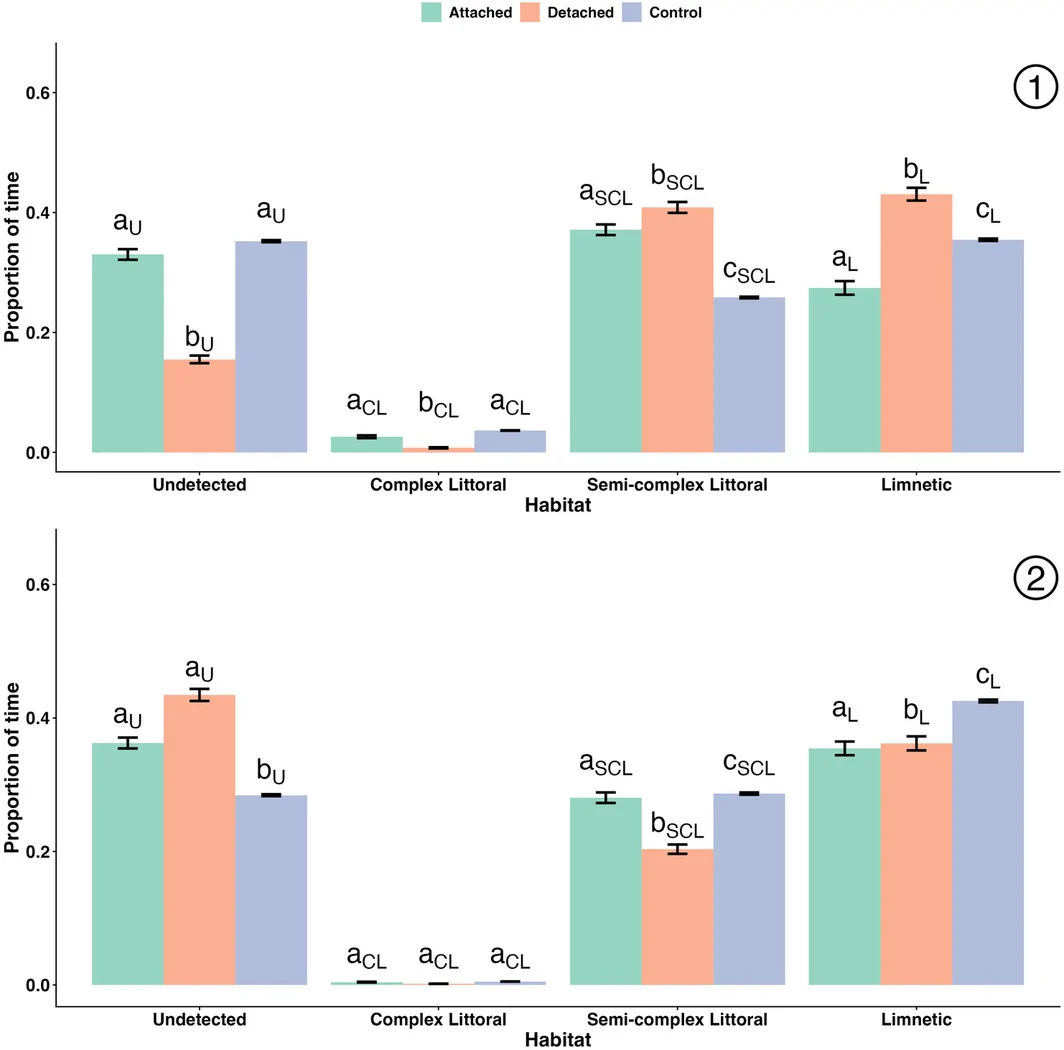
Proportion of time (± standard error) that Largemouth Bass *Micropterus nigricans* (1) and Northern Pike *Esox lucius* (2) spent in different habitats based on their pop‐off biologging status (i.e., attached, detached or control). Dissimilar letters represent significant differences in the proportion of time spent in a habitat. Subscripts on the letters are used to represent which habitat group the letters belong to.

Pop‐off biologging package status influenced the proportion of time that Largemouth Bass spent in different habitats (χ^2^ = 498.060, df = 6, *p* < 0.001). Habitat use of Largemouth Bass shifted substantially once the pop‐off biologging packages were detached (Table 1; Figure 6‐1). Generally, when pop‐off biologging packages were attached to Largemouth Bass, they spent more time near refuge compared to the controls or when the tags were detached (Figure 6‐1). Largemouth Bass spent significantly more time in the limnetic and semi‐complex littoral zone once pop‐off biologging packages were detached, compared to when they were attached (Table 1; Figure 6‐1). Similarly, control Largemouth Bass spent more time in the limnetic habitat and less time in the semi‐complex habitat compared to when the pop‐off biologging packages were attached. When equipped with a pop‐off biologger, Largemouth Bass went undetected at a similar rate as the control Largemouth Bass, but was significantly more than when the pop‐off biologging packages were detached (Table 1; Figure 6‐1).

**TABLE 1:** Pairwise comparisons of the proportion of time spent in habitats between the different periods of pop‐off biologging attachment of Largemouth Bass *Micropterus nigricans* and Northern Pike *Esox lucius*.

Species	Habitat	Comparison	*z* ratio	Standard error (SE)	*p*‐value
*Largemouth Bass*	Undetected	**Attached–detached**	**10.143**	**0.074**	**<0.001**
		Attached**–**control	1.388	0.038	0.347
		**Detached–control**	**−12.315**	**0.022**	**<0.001**
	Complex littoral	Attached–detached	2.581	0.048	0.027
		Attached–control	−1.651	0.032	0.225
		Detached–control	−5.130	0.028	<0.001
	Semi‐ complex littoral	**Attached–detached**	**−6.250**	**0.036**	**<0.001**
		**Attached–control**	**3.188**	**0.041**	**0.004**
		**Detached–control**	**11.516**	**0.055**	**<0.001**
	Limnetic	**Attached–detached**	**−11.519**	**0.027**	**<0.001**
		**Attached–control**	**−5.609**	**0.029**	**<0.001**
		**Detached–control**	**9.607**	**0.051**	**<0.001**
*Northern Pike*	Undetected	Attached–detached	−1.800	0.042	0.170
		**Attached–control**	**4.726**	**0.048**	**<0.001**
		**Detached–control**	**5.537**	**0.054**	**<0.001**
	Complex littoral	Attached–detached	0.397	0.042	0.917
		Attached–control	−0.064	0.038	0.998
		Detached–control	−0.488	0.033	0.877
	Semi‐complex littoral	**Attached–detached**	**3.803**	**0.052**	**<0.001**
		Attached–control	−0.541	0.038	0.851
		**Detached–control**	**−4.271**	**0.033**	**<0.001**
	Limnetic	**Attached–detached**	**−3.478**	**0.039**	**0.002**
		**Attached–control**	**−8.114**	**0.027**	**<0.001**
		**Detached–control**	**−3.900**	**0.035**	**<0.001**

*Note*: Bold rows represent comparisons that are significantly different.

**FIGURE 7:**
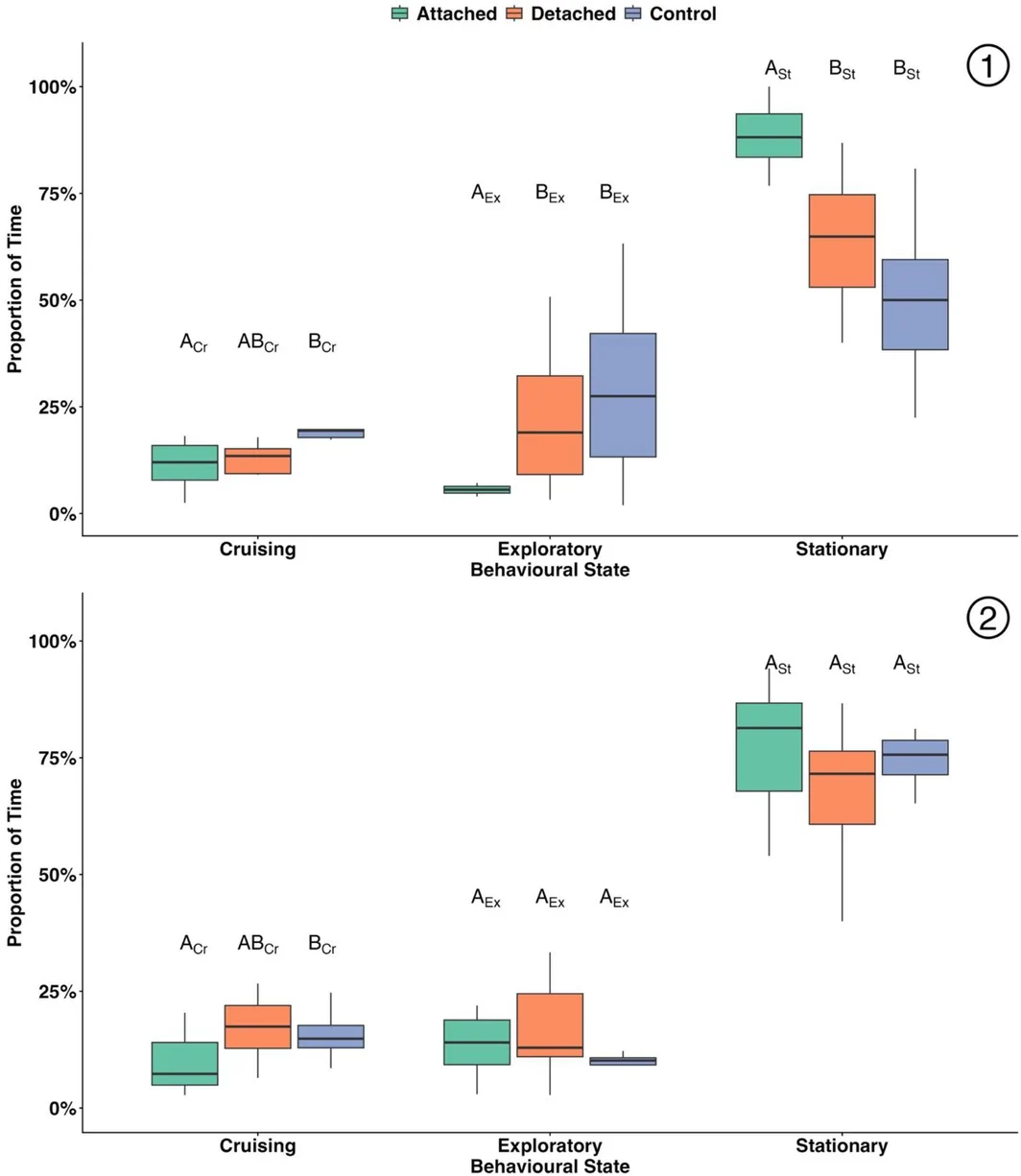
Proportion of time that Largemouth Bass *Micropterus nigricans* (1) and Northern Pike *Esox lucius* (2) spent engaging in the different behaviour types (cruising, exploring, stationary) while pop‐off biologging packages were attached (72 h), after they were detached (72 h) and controls that were not equipped with pop‐off biologging packages (144 h). Behaviour types were classified with k‐means clustering, whereby cruising behaviour was identified with long linear movements, while exploratory behaviour consisted of long step lengths, with high tortuosity and smaller linear distances. Short step lengths and small net displacement were classified as stationary behaviour. Dissimilar letters represent significant differences in the proportion of time engaging in the specific behaviour. Subscripts on the letters are used to represent which behaviour type the letters belong to.

Movement behaviour analyses revealed clear differences between control Largemouth Bass and the Largemouth Bass that were attached with pop‐off biologging packages. The movement behaviour of Largemouth Bass changed once the pop‐off biologging packages were detached and was similar to that of the control Largemouth Bass once detached (Figure 7‐1). The pop‐off biologging package status had a significant influence on the cruising behaviour (χ^2^ = 55.534, df = 2, *p* < 0.001), exploration behaviour (χ^2^ = 9.350, df = 2, *p* = 0.009) and stationary behaviour (χ^2^ = 34.211, df = 2, *p* < 0.001) of Largemouth Bass. Control Largemouth Bass spent significantly more time cruising (53% more) compared to Largemouth Bass attached with pop‐off biologging packages, and control Largemouth Bass spent significantly less time stationary (Table 2; Figure 7‐1). Stationary behaviour was reduced by 51% when pop‐off biologging packages were detached from Largemouth Bass compared to when the pop‐off biologging packages were attached. Largemouth Bass also spent 71% more time stationary when attached with a pop‐off biologging package compared to controls. The exploration behaviour of Largemouth Bass increased significantly after the pop‐off biologging packages were detached (increase of 96%), and when attached with the pop‐off biologgers, they spent significantly less time (97% less) exploring compared to controls. Once the pop‐off biologging packages were detached, Largemouth Bass behaved similar to control Largemouth Bass (Table 2, Figure 7‐1).

**TABLE 2:** Pairwise comparisons of movement behaviour types across the pop‐off biologging package status (e.g., attached, detached and control) for Largemouth Bass *Micropterus nigricans*.

Species	Behaviour type	Comparison	*z* ratio	SE	*p*‐value
*Largemouth Bass*	Cruising	Attached–detached	−2.139	0.148	0.082
		**Attached–control**	**−3.032**	**0.120**	**0.007**
		Detached–control	−1.297	0.178	0.397
	Exploratory	**Attached–detached**	**−5.829**	**0.025**	**<0.001**
		**Attached–control**	**−4.024**	**0.034**	**<0.001**
		Detached–control	−0.141	0.557	0.989
	Stationary	**Attached–detached**	**7.075**	**1.160**	**<0.001**
		**Attached–control**	**5.074**	**3.340**	**<0.001**
		Detached–control	1.245	0.608	0.427

*Note*: Bold rows represent comparisons that are significantly different.

### 3.2 Northern pike

Pop‐off biologging package status influenced the proportion of time that Northern Pike spent in different habitats (χ^2^ = 236.599, df = 6, *p* < 0.001). Habitat used by Northern Pike changed significantly once the pop‐off biologging packages were detached (Table 1; Figure 6‐2). When Northern Pike were equipped with a pop‐off biologging package, they spent a similar amount of time in the undetected state as when the pop‐off biologging packages were detached. However, control Northern Pike spent significantly less time undetected compared to when the tags were attached or detached (Table 1). Control Northern Pike spent significantly more time in the limnetic and semi‐complex habitats compared to when the pop‐off biologging packages were attached or detached. Once the pop‐off biologgers were detached, Northern Pike spent significantly less time in the semi‐complex habitat and more time in the limnetic habitat compared to when the pop‐off biologging packages were attached (Table 1, Figure 6‐2). Finally, pop‐off biologging packages did not significantly influence the movement behaviour of Northern Pike. Neither the exploratory behaviour (χ^2^ = 5.596, df = 2, *p* = 0.061), stationary behaviour (χ^2^ = 0.244, df = 2, *p* = 0.885), or the cruising behaviour (χ^2^ = 4.319, df = 2, *p* = 0.115) of Northern Pike differed when equipped with pop‐off biologging packages compared to the controls or when the packages were detached (Figure 7‐2).

## 4 DISCUSSION

Although there have been studies that have assessed the impacts of external tagging (reviewed in Jepsen et al., 2015), fewer have assessed the behaviour of fish in the wild with external biologging packages. We used fine‐scale positions of fish that were previously tagged with acoustic transmitters, equipped with external pop‐off biologging packages and compared their behaviour with and without those external biologgers. This unique opportunity to assess tag effects on the behaviour of fish provides valuable insight that is worth considering in future studies using external tagging methods when assessing the behaviour of fish in the wild. Overall, we found clear evidence that external pop‐off biologging packages influenced the behaviour and habitat use of both Largemouth Bass, and only habitat use of Northern Pike. Largemouth Bass spent greater time in habitats that were more complex and provided more refuge when attached with an external pop‐off biologging package. Once the external pop‐off biologging packages were detached from the Largemouth Bass, they spent more time in the limnetic zone of the lake. Largemouth Bass spent more time stationary when equipped with an external pop‐off biologging package, and only after the pop‐off package was detached did they spend more time cruising and exploring, similar to controls. When equipped with pop‐off biologging packages, Northern Pike spent more time in the semi‐complex habitat compared to after the tagging package detached. Interestingly, control Northern Pike spent even more time in the semi‐complex habitat compared to when the pop‐off packages were attached, possibly suggesting that the influence of pop‐off packages on the normal behaviour of Northern Pike is not as biologically meaningful. The overall movement behaviour of Northern Pike was unaffected by the attachment of the external pop‐off biologging package.

Habitat complexity has an important influence on the ability to acquire food and seek refuge from predators (Mikheev et al., 1997; Mikheev et al., 2010). The habitat type used when the external pop‐off biologging package were removed suggests that the fish may feel they are at greater risk of predation (e.g., from mammals or avian predators) when the external pop‐off biologging packages are attached and feel more ‘comfortable’ to explore open habitats once the pop‐off biologging packages are detached. Feeding efficacy increases with decreasing habitat complexity (Eklöv, 1997; Savino & Stein, 1982), suggesting that being able to use the open limnetic habitat frequently is important for food acquisition. The shift in habitat used with and without an external pop‐off biologging package is an indication that behavioural trends changed from vulnerable, frightened or defensive in the complex habitat areas to more of a risk‐prone behaviour (e.g., bold and exploratory) where they were actively searching for forage in the limnetic zones. Although fish are able to feed in semi‐ to complex habitats, the efficacy of capturing prey is typically reduced (Eklöv, 1997; Savino & Stein, 1982). Ultimately, anything that could reduce the feeding efficacy of fish by not engaging in actively seeking prey (e.g., equipped with the external pop‐off biologging package) could have negative implications for the fitness of fish.

External tagging methods have the potential to create excessive drag that can impact swimming performance, energy expenditure and oxygen consumption of fish (reviewed in Jepsen et al., 2015). Tag burden from external tagging methods reduces critical swimming speeds of fish (McCleave & Stred, 1975; Peake et al., 1997), decreases time to exhaustion (Mellas & Haynes, 1985) and increases oxygen consumption (Steinhausen et al., 2006). However, the influence of tag burden is size‐specific, and not all fish are impacted to the same degree (Gray & Haynes, 1979; Sundström & Gruber, 2002). There was evidence that the external pop‐off biologging packages used in this study had an impact on the movement of Largemouth Bass, whereas the movement of Northern Pike was not influenced. This is likely due to Northern Pike having a larger body size compared to Largemouth Bass, and thus could accommodate the extra drag from the external pop‐off biologging package. The increase in cruising and exploratory behaviour of Largemouth Bass after the external pop‐off biologging packages were detached suggests that their swimming performance might have been influenced by the attachment of the external pop‐off biologging packages. Largemouth Bass likely needed to expend more energy to move around the environment and became exhausted faster when equipped with external pop‐off biologging packages. Finally, swimming performance is vital for avoiding or evading predators (Walker et al., 2005) and capturing prey (Borla et al., 2002; Budick & O'Malley, 2000; Higham et al., 2007; Rice & Westneat, 2005). Therefore, negative impacts on fitness could occur for Largemouth Bass when external pop‐off biologging packages are given, as the swimming performance (i.e., movement behaviour) of fish was impeded.

Future research should aim to identify the body to external tag ratio or other aspects of tag design (e.g., shape influences on drag) that do not cause biologically meaningful differences in the behaviour of fishes, building on the existing evidence base (see Jepsen et al., 2015). The current study suggests that larger‐bodied fish (e.g., Northern Pike) were marginally impacted by the attachment of the external pop‐off biologging packages, yet the results do not provide evidence of how larger fish in marine systems or migratory species would be impacted by the attachment of external biologging packages. With such small nuances in the behaviour of Northern Pike in our study, it is unlikely that these packages would have much of an effect on larger fish species, although that has yet to be tested. The approach we used to test tagging impacts associated with external attachment was unique and took advantage of a small, high‐resolution whole‐lake telemetry array capable of positioning tagged fish with high levels of precision and accuracy. Similar arrays are not overly common, such that it is not often that the approach used here could be applied elsewhere. Nonetheless, it has merit where such opportunities exist.

As more studies use external biologging methods to assess the behaviour of fish in the wild, it is important that the interpretation of the results considers the impacts that biologgers have on the habitat use and movement behaviour of fish. We found that habitat use and movement behaviour of fish differed when equipped with external pop‐off biologging packages. The swimming activity of Largemouth Bass is likely being underestimated when using external tagging methods, while the swimming activity of Northern Pike is presumably more accurate. Because habitat use was influenced by the presence of the external pop‐off biologging package, determining if fish are using those habitats because of an acute stress (e.g., catch‐and‐release) event will be challenging, but critical for determining the extent of the acute stress event. Being able to disentangle tagging effects from acute stress‐induced behavioural differences will be important for the interpretation and contextualization of results in future studies. Finally, given the changes in habitat use and movement behaviour that occur from carrying external pop‐off biologging packages, unnecessarily long attachment periods should be avoided. In similar contexts to this study (e.g., fish of small to moderate body size), external pop‐off biologging packages should only be attached to fish for the period of time that fish are behaviourally impaired from the capture, or an acute stress event, to avoid any negative impacts the pop‐off biologging packages could have on the fitness of fish.

## AUTHOR CONTRIBUTIONS

Conceptualization: L.L., S.J.C.; data curation: L.L., M.B.S., G.D.R.; data collection: L.L., M.B.S., G.D.R.; formal analysis: L.L., G.D.R., J.W.B.; visualization: L.L., G.D.R., J.W.B.; funding acquisition: S.J.C., G.D.R., A.T.F.; writing—original draft: L.L.; and writing—review & editing: L.L., S.J.C. A.J.D., G.D.R., J.W.B., M.B.S., A.T.F.

## CONFLICT OF INTEREST STATEMENT

The authors declare no conflicts of interest.

## Data Availability

The data that support the findings of this study are available from the corresponding author upon reasonable request.

## References

[jfb70560-bib-0001] Algera, D. A. , Brownscombe, J. W. , Gilmour, K. M. , Lawrence, M. J. , Zolderdo, A. J. , & Cooke, S. J. (2017). Cortisol treatment affects locomotor activity and swimming behavior of male smallmouth bass engaged in paternal care: A field study using acceleration biologgers. Physiology & Behavior, 181, 59–68. 10.1016/j.physbeh.2017.08.026 28866027

[jfb70560-bib-0079] Appelhans, T. , Russell, K. , & Busetto, L. (2026). mapedit: Interactive Editing of Spatial Data in R. CRAN: Contributed Packages. The R Foundation.

[jfb70560-bib-0003] Bates, D. , Mächler, M. , Bolker, B. , & Walker, S. (2015). Fitting linear mixed‐effects models using lme4. Journal of Statistical Software, 67(1), 1–48.

[jfb70560-bib-0004] Beitinger, T. L. (1990). Behavioral reactions for the assessment of stress in fishes. Journal of Great Lakes Research, 16, 495–528.

[jfb70560-bib-0005] Benhamou, S. (2004). How to reliably estimate the tortuosity of an animal's path: Straightness, sinuosity, or fractal dimension? Journal of Theoretical Biology, 229(1), 209–220. 10.1016/j.jtbi.2004.03.016 15207476

[jfb70560-bib-0006] Borla, M. A. , Palecek, B. , Budick, S. , & O'Malley, D. M. (2002). Prey capture by larval zebrafish: Evidence for fine axial motor control. Brain, Behavior and Evolution, 60(4), 207–229. 10.1159/000066699 12457080

[jfb70560-bib-0007] Boudreau, M. R. , Rainbolt, S. , Lister, H. , Diamond, A. , Lee, A. M. , & Allen, P. J. (2025). Biologger attachment and retention in channel catfish (*Ictalurus punctatus*). Aquaculture, 601, 742281.

[jfb70560-bib-0008] Bovet, P. , & Benhamou, S. (1988). Spatial analysis of animals' movement using a correlated random walk model. Journal of Theoretical Biology, 131, 419–433.

[jfb70560-bib-0009] Brewster, L. R. , Ibrahim, A. K. , DeGroot, B. C. , Ostendorf, T. J. , Zhuang, H. , Chérubin, L. M. , & Ajemian, M. J. (2021). Classifying goliath grouper (*Epinephelus itajara*) behaviors from a novel, multi‐sensor tag. Sensors, 21(19), 6392. 10.3390/s21196392 PMC8512029 34640710

[jfb70560-bib-0010] Brooks, M. E. , Kristensen, K. , van Benthem, K. J. , Magnusson, A. , Berg, C. W. , Nielsen, A. , Skaug, H. J. , Maechler, M. , & Bolker, B. M. (2017). glmmTMB balances speed and flexibility among packages for zero‐inflated generalized linear mixed modeling. The R Journal, 9(2), 378–400.

[jfb70560-bib-0011] Brownsmith, C. B. (1977). Foraging rates of starlings in two habitats. The Condor, 79, 386–387.

[jfb70560-bib-0012] Budaev, S. V. (1997). Alternative styles in the European wrasse, *Symphodus ocellatus* – Boldness‐related schooling tendency. Environmental Biology of Fishes, 49, 71–78.

[jfb70560-bib-0013] Budick, S. A. , & O'Malley, D. M. (2000). Locomotor repertoire of the larval zebrafish: Swimming, turning and prey capture. Journal of Experimental Biology, 203(17), 2565–2579. 10.1242/jeb.203.17.2565 10934000

[jfb70560-bib-0014] Clark, T. D. , Sandblom, E. , Hinch, S. G. , Patterson, D. A. , Frappell, P. B. , & Farrell, A. P. (2010). Simultaneous biologging of heart rate and acceleration, and their relationships with energy expenditure in free‐swimming sockeye salmon (*Oncorhynchus nerka*). Journal of Comparative Physiology B, 180(5), 673–684. 10.1007/s00360-009-0442-5 20063165

[jfb70560-bib-0016] Cooke, S. J. , Bergman, J. N. , Twardek, W. M. , Piczak, M. L. , Casselberry, G. A. , Lutek, K. , Dahlmo, L. S. , Birnie‐Gauvin, K. , Griffin, L. P. , Brownscombe, J. W. , Raby, G. D. , Standen, E. M. , Hordodysky, A. Z. , Johnsen, S. , Danylchuk, A. J. , Furey, N. B. , Gallagher, A. J. , Lédée, E. J. I. , Midwood, J. D. , … Lennox, R. J. (2022). The movement ecology of fishes. Journal of Fish Biology, 101, 756–779. 10.1111/jfb.15153 35788929

[jfb70560-bib-0015] Cooke, S. , Hinch, S. G. , Lucas, M. C. , & Lutcavage, M. (2012). Biotelemetry and biologging. In Fisheries techniques (3rd ed., pp. 819–860). American Fisheries Society.

[jfb70560-bib-0017] Cooke, S. J. , Messmer, V. , Tobin, A. J. , Pratchett, M. S. , & Clark, T. D. (2014). Refuge‐seeking impairments Mirror metabolic recovery following fisheries‐related stressors in the Spanish flag snapper (*Lutjanus carponotatus*) on the great barrier reef. Physiological and Biochemical Zoology, 87, 136–147. 10.1086/671166 24457928

[jfb70560-bib-0018] Danylchuk, A. J. , LaRochelle, L. , Raby, G. D. , Bennett, B. , Cooke, S. J. , & Brownscombe, J. W. (2026). Hooking injury, physiological stress, and post‐release activity patterns of giant trevally (*Caranx ignobilis*) captured using spinning gear in a catch‐and‐release recreational fishing. Environmental Biology of Fishes, 109, 45.

[jfb70560-bib-0019] Danylchuk, S. E. , Danylchuk, A. J. , Cooke, S. J. , Goldberg, T. L. , Koppelman, J. , & Philipp, D. P. (2007). Effects of recreational angling on the post‐release behavior and predation of bonefish (*Albula vulpes*): The role of equilibrium status at the time of release. Journal of Experimental Marine Biology and Ecology, 346, 127–133.

[jfb70560-bib-0020] Echave, K. B. (2016). Feasibility of tagging sablefish, *Anoplopoma fimbria*, with pop‐off satellite tags in the Northeast Pacific Ocean. NOAA Technical Memorandum *NMFS‐AFSC‐320*.

[jfb70560-bib-0021] Eklöv, P. (1997). Effects of habitat complexity and prey abundance on the spatial and temporal distributions of perch (*Perca fluviatilis*) and pike (*Esox lucius*). Canadian Journal of Fisheries and Aquatic Sciences, 54, 1520–1531.

[jfb70560-bib-0022] Elliot, C. W. , Ridgway, M. S. , Blanchfield, P. J. , & Tufts, B. L. (2023). Novel insights gained from tagging walleye (*Sander vitreus*) with pop‐off data storage tags and acoustic transmitters in Lake Ontario. Journal of Great Lakes Research, 49(2), 515–530.

[jfb70560-bib-0023] Enefalk, Å. , & Bergman, E. (2016). Effect of fine wood on juvenile brown trout behaviour in experimental stream channels. Ecology of Freshwater Fish, 25(4), 664–673.

[jfb70560-bib-0024] Gray, R. H. , & Haynes, J. M. (1979). Spawning migration of adult chinook salmon (*Oncorhynchus tshawytscha*) carrying external and internal radio transmitters. Journal of the Fisheries Research Board of Canada, 36, 1060–1064.

[jfb70560-bib-0025] Hedger, R. D. , Rikardsen, A. H. , & Thorstad, E. B. (2017). Pop‐up satellite archival tag effects on diving behaviour, growth and survival of adult Atlantic salmon *Salmo salar* at sea. Journal of Fish Biology, 90, 294–310. 10.1111/jfb.13174 27917476

[jfb70560-bib-0026] Higham, T. E. , Hulsey, C. D. , Říčan, O. , & Carroll, A. M. (2007). Feeding with speed: Prey capture evolution in cichlids. Journal of Evolutionary Biology, 20(1), 70–78. 10.1111/j.1420-9101.2006.01227.x 17210001

[jfb70560-bib-0027] Huntingford, F. A. , Adams, C. , Braithwaite, V. A. , Kadri, S. , Pottinger, T. G. , Sandøe, P. , & Turnbull, J. F. (2006). Current issues in fish welfare. Journal of Fish Biology, 68, 332–372.

[jfb70560-bib-0028] Hussey, N. E. , Kessel, S. T. , Aarestrup, K. , Cooke, S. J. , Cowley, P. D. , Fisk, A. T. , Harcourt, R. G. , Holland, K. N. , Iverson, S. J. , Kocik, J. F. , Flemming, J. E. M. , & Whoriskey, F. G. (2015). Aquatic animal telemetry: A panoramic window into the underwater world. Science, 348(6240), 1255642. 10.1126/science.1255642 26068859

[jfb70560-bib-0029] Iwama, G. K. , Pickering, A. D. , Sumpter, J. P. , & Schreck, C. B. (1997). Fish stress and health in aquaculture. Cambridge University Press.

[jfb70560-bib-0030] Janak, J. M. , Brown, R. S. , Colotelo, A. H. , Pflugrath, B. D. , & Stephenson, J. R. (2012). The effects of neutrally buoyant, externally attached transmitters in swimming performance and predator avoidance of juvenile Chinook salmon. Transactions of the American Fisheries Society, 141, 1424–1432.

[jfb70560-bib-0031] Jepsen, N. , Thorstad, E. B. , Havn, T. , & Lucas, M. C. (2015). The use of external electronic tags on fish: An evaluation of tag retention and tagging effects. Animal Biotelemetry, 3, 49.

[jfb70560-bib-0081] LaRochelle, L. , Bernardi, J. , Madden, J. C. , Brownscombe, J. W. , Goodenough, A. M. , Danylchuk, A. J. , & Cooke, S. J. (2025). Effects of recreational fishing gear type on reflex impairment and post‐release swimming activity of smallmouth bass. Fisheries Management and Ecology, 32(2), e12776. 10.1111/fme.12776

[jfb70560-bib-0032] LaRochelle, L. , Burton, D. , Madden, J. C. , Danylchuk, S. C. , Cooke, S. J. , & Danylchuk, A. J. (2023). Using a novel biologging approach to assess how different handling practices influence the post‐release behaviour of northern pike across a wide range of body sizes. Aquatic Living Resources, 36(25), 25.

[jfb70560-bib-0033] LaRochelle, L. , Chhor, A. D. , Brownscombe, J. W. , Zolderdo, A. J. , Danylchuk, A. J. , & Cooke, S. J. (2021). Ice‐fishing handling practices and their effects of the short‐term post‐release behaviour of largemouth bass. Fisheries Research, 243, 106084.

[jfb70560-bib-0034] LaRochelle, L. , Griffin, L. P. , Brownscombe, J. W. , Elvidge, C. K. , Hasler, C. T. , Robichaud, J. A. , Madden, J. C. , Landsman, S. J. , Raoult, R. , Danylchuk, A. J. , & Cooke, S. J. (2025). On the behaviour of fish released following fisheries capture: Methods, endpoints, and consequences. Reviews in Fisheries Science & Aquaculture, 33(4), 629–656.

[jfb70560-bib-0035] LaRochelle, L. , Haniford, L. , Burton, D. , Bieber, J. F. , Robichaud, J. A. , Suski, C. D. , Danylchuk, A. J. , & Cooke, S. J. (2024). Do live‐well additives influence the physiology and behavioral recovery of largemouth bass? North American Journal of Fisheries Management, 44(1), 189–203.

[jfb70560-bib-0036] LaRochelle, L. , Shorgan, M. , Raby, G. D. , Brownscombe, J. W. , Howell, B. E. , Fisk, A. T. , Danylchuk, A. J. , & Cooke, S. J. (2026). A field study combining biotelemetry and biologging to determine how long the behaviour of fish is impacted by catch‐and‐release angling. Canadian Journal of Fisheries and Aquatic Sciences, 83, 1–15.

[jfb70560-bib-0037] Lear, K. O. , & Whitney, N. M. (2016). Bringing data to the surface: Recovering data loggers for large sample sizes from marine vertebrates. Animal Biotelemetry, 4, 12.

[jfb70560-bib-0038] Lenth, R. (2025). emmeans: Estimated marginal means, aka least‐squares means.

[jfb70560-bib-0039] Louison, M. J. , LaRochelle, L. , & Cooke, S. J. (2023). Effectiveness of barotrauma mitigation methods in ice‐angled bluegill and black crappie. Fisheries Management and Ecology, 30(3), 229–239.

[jfb70560-bib-0040] Lynch, S. D. , Marcek, B. J. , Marshall, H. M. , Bushnell, P. G. , Bernal, D. , & Brill, R. W. (2017). The effects of pop‐up satellite archival tags (PSATs) on the metabolic rate and swimming kinematics of juvenile sandbar shark *Carcharhinus plumbeus* . Fisheries Research, 186(1), 205–215.

[jfb70560-bib-0041] Madden, J. C. , LaRochelle, L. , Goodenough, A. M. , Danylchuk, A. J. , & Cooke, S. J. (2025). Free falling: Fizzing wild‐caught smallmouth bass results in the inability to control buoyancy in deep water. Fisheries Management and Ecology, 32(4), 41–49.

[jfb70560-bib-0042] Mäkinen, T. S. , Niemelä, E. , Moen, K. , & Lindström, R. (2000). Behaviour of gill‐net and rod‐captured Atlantic salmon (*Salmo salar* L.) during upstream migration and following radio tagging. Fisheries Research, 45, 117–127.

[jfb70560-bib-0043] McCleave, J. D. , & Stred, K. A. (1975). Effect of dummy telemetry transmitters on stamina of Atlantic salmon (*Salmon salar*) smolts. Journal of the Fisheries Research Board of Canada, 32, 559–563.

[jfb70560-bib-0044] Mellas, E. J. , & Haynes, J. M. (1985). Swimming performance and behavior of rainbow trout (*Salmo gairdneri*) and white perch (*Morone americana*): Effects of attaching telemetry transmitters. Canadian Journal of Fisheries and Aquatic Sciences, 42, 488–493.

[jfb70560-bib-0045] Methling, C. , Tudorache, C. , Skov, P. V. , & Steffensen, J. F. (2011). Pop up satellite tags impair swimming performance and energetics of the European eel (*Anguila anguila*). PLoS One, 6(6), e20797. 10.1371/journal.pone.0020797 PMC3110781 21687674

[jfb70560-bib-0046] Mikheev, V. N. , Afonina, M. O. , & Gaisina, E. V. (1997). Visually heterogenous environment stimulates foraging activity cichlids. Journal of Ichthyology, 37(1), 93–97.

[jfb70560-bib-0047] Mikheev, V. N. , Afonina, M. O. , & Pavlov, D. S. (2010). Habitat heterogeneity and fish behavior: Units of heterogeneity as a resource and as a source of information. Journal of Ichthyology, 50, 386–395.

[jfb70560-bib-0048] O'Neill, R. , Maoiléidigh, Ó. N. , McGinnity, P. , Bond, N. , & Culloty, S. (2018). The novel use of pop‐off satellite tags (PSATs) to investigate the migratory behaviour of European sea bass *Dicentrarchus labrax* . Journal of Fish Biology, 92(5), 1404–1421. 10.1111/jfb.13594 29607514

[jfb70560-bib-0049] Peake, S. , McKinley, R. S. , Scruton, D. A. , & Moccia, R. (1997). Influence of transmitter attachment procedures on swimming performance of wild and hatchery‐reared Atlantic salmon smolts. Transactions of the American Fisheries Society, 126, 707–714.

[jfb70560-bib-0050] Posit team . (2025). RStudio: Integrated development environment for R. Posit Software, PBC.

[jfb70560-bib-0051] Postlethwaite, C. M. , Brown, P. , & Dennis, T. E. (2013). A new multi‐scale measure for analysing animal movement. Journal of Theoretical Biology, 317, 175–185. 10.1016/j.jtbi.2012.10.007 23079283

[jfb70560-bib-0053] R Core Team . (2025). R: A language and environment for statistical computing. R Foundation for Statistical Computing.

[jfb70560-bib-0054] Raby, G. D. , Johnson, T. B. , Kessel, S. T. , Stewart, T. J. , & Fisk, A. T. (2017). A field test of the use of pop‐off data storage tags in freshwater fishes. Journal of Fish Biology, 91(6), 1623–1641. 10.1111/jfb.13476 29023720

[jfb70560-bib-0055] Radabaugh, N. B. , Bauer, W. F. , & Brown, M. L. (2010). A comparison of seasonal movement patterns of yellow perch in simple and complex lake basins. North American Journal of Fisheries Management, 30(1), 179–190.

[jfb70560-bib-0056] Reeve, C. , LaRochelle, L. , Bihun, C. J. , Brownscombe, J. W. , & Cooke, S. J. (2025). Winter behaviour and energetics of free‐swimming largemouth bass. Canadian Journal of Fisheries and Aquatic Sciences, 82, 1–16.

[jfb70560-bib-0057] Reid, C. H. , Crossman, J. A. , Marrello, M. M. , LaRochelle, L. , & Cooke, S. J. (2025). Quantifying behavioural impairment as a proxy for physiological stress to improve welfare of imperilled white sturgeon (*Acipenser transmontanus*) during routine sampling efforts. Applied Animal Behaviour Science, 292, 106762.

[jfb70560-bib-0058] Reid, C. H. , LaRochelle, L. , Madden, J. C. , Haniford, L. E. , Burton, D. , Midwood, J. D. , & Cooke, S. J. (2024). Relative effects of electro‐immobilisation and chemical anaesthesia on *in situ* post‐release behaviour and reproductive success in nesting smallmouth bass (*Micropterus dolomieu*). Applied Animal Behaviour Science, 273, 106239.

[jfb70560-bib-0059] Reid, C. H. , Vandergoot, C. S. , Midwood, J. D. , Stevens, E. D. , Bowker, J. , & Cooke, S. J. (2019). On the Electroimmobilization of fishes for research and practice: Opportunities, challenges, and research needs. Fisheries, 44, 576–585.

[jfb70560-bib-0060] Rice, A. N. , & Westneat, M. W. (2005). Coordination of feeding, locomotor and visual systems in parrotfishes (Teleostei: Labridae). Journal of Experimental Biology, 208(18), 3503–3518. 10.1242/jeb.01779 16155223

[jfb70560-bib-0061] Robichaud, J. A. , Haley, A. L. , LaRochelle, L. , Dello Russo, J. , Zhang, J. , Cunningham, K. E. , Lawson, L. , Bergman, J. N. , Jolin, E. , Madden, J. C. , Matley, J. , Klinard, N. V. , Barbosa Martins, A. , Kessel, S. T. , Huveneers, C. , Stokesbury, M. , Hussey, N. E. , Cooke, S. J. , & Piczak, M. (2025). Global trends in aquatic animal satellite telemetry studies. Environmental Reviews, 33, 1–19.

[jfb70560-bib-0062] Savino, J. F. , & Stein, R. A. (1982). Predator‐prey interaction between fish predators and their prey: Effects of plant density. Animal Behaviour, 37, 11–321.

[jfb70560-bib-0063] Schooley, R. L. , Sharpe, P. B. , & Van Horne, B. (1996). Can shrub cover increase predation risk for desert rodent? Canadian Journal of Zoology, 74, 157–163.

[jfb70560-bib-0080] Schreck, C. B. , Olla, B. L. , & Davis, M. W. (1997). Behavioral responses to stress. In G. K. Iwama , A. D. Pickering , J. P. Sumpter , & C. B. Schreck (Eds.), Fish stress and health in aquaculture (Society for Experimental Biology seminar series) (Vol. 62, pp. 145–170). Cambridge University Press.

[jfb70560-bib-0065] Shorgan, M. B. , Howell, B. E. , LaRochelle, L. , Cooke, S. J. , Fisk, A. T. , Vandergoot, C. S. , & Raby, G. D. (2025). Behavioural impacts of MS‐222 and electro‐immobilization on wild fish assessed using a whole lake telemetry system. Canadian Journal of Fisheries and Aquatic Sciences, 82, 1–12.

[jfb70560-bib-0066] Shorgan, M. B. , Raby, G. D. , Fedus, A. L. , Howell, B. E. , Haniford, L. S. E. , Howitt, L. C. , Klinard, N. V. , Matley, J. K. , Brownscombe, J. W. , Cooke, S. J. , & Fisk, A. (2026). Effects of surgical implantation of electronic tags in fishes: A review and meta‐analysis. Reviews in Fish Biology and Fisheries, 36, 14.

[jfb70560-bib-0067] Shorter, K. A. , Murray, M. M. , Johnson, M. , Moore, M. , & Howle, L. E. (2014). Drag of suction cup tags on swimming animals: Modeling and measurement. Marine Mammal Science, 30(2), 726–746.

[jfb70560-bib-0068] Smith, F. (2013). Understanding HPE in the VEMCO positioning system (VPS). Document #: DOC‐005457‐01.

[jfb70560-bib-0069] Steinhausen, M. F. , Andersen, N. G. , & Steffensen, J. F. (2006). The effect of external dummy transmitters on oxygen consumption and performance of swimming Atlantic cod. Journal of Fish Biology, 69, 951–956.

[jfb70560-bib-0070] Sundström, L. F. , & Gruber, S. H. (2002). Effects of capture and transmitter attachments on the swimming speed of large juvenile lemon sharks in the wild. Journal of Fish Biology, 61, 834–838.

[jfb70560-bib-0078] Thompson, L. A. , Cooke, S. J. , Donaldson, M. R. , Hanson, K. C. , Gingerich, A. , Klefoth, T. , & Arlinghaus, R. (2008). Physiology, behavior, and survival of angled and air‐exposed largemouth bass. North American Journal of Fisheries Management, 28(4), 1059–1068. 10.1577/m07-079.1

[jfb70560-bib-0071] Tudorache, C. , Burgerhout, E. , Brittijn, S. , & van den Thillart, G. (2014). The effect of drag and attachment site of tags on swimming eels: Experimental quantification and evaluation tool. PLoS One, 4(9), e112280. 10.1371/journal.pone.0112280 PMC4237349 25409179

[jfb70560-bib-0072] Walker, J. A. , Ghalambor, C. K. , Griset, O. L. , McKenney, D. , & Reznick, D. N. (2005). Do faster starts increase the probability of evading predators? Functional Ecology, 19(5), 808–815.

[jfb70560-bib-0073] Whitney, N. M. , White, C. F. , Gleiss, A. C. , Schwieterman, G. D. , Anderson, P. , Hueter, R. E. , & Skomal, G. B. (2016). A novel method for determining post‐release mortality, behavior, and recovery period using acceleration data loggers. Fisheries Research, 183, 210–221.

[jfb70560-bib-0074] Wickham, H. (2016). Ggplot2: Elegant graphics for data analysis. Springer‐Verlag.

[jfb70560-bib-0075] Williams, H. J. , Taylor, L. A. , Benhamou, S. , Bijleveld, A. I. , Clay, T. A. , de Grissac, S. , Demšar, U. , English, H. M. , Franconi, N. , Gómez‐Laich, A. , Griffiths, R. C. , Kay, W. P. , Morales, J. M. , Potts, J. R. , Rogerson, K. F. , Rutz, C. , Spelt, A. , Trvail, A. M. , Wilson, R. P. , & Börger, L. (2020). Optimizing the use of biologgers for movement ecology research. Journal of Animal Ecology, 89(1), 186–206. 10.1111/1365-2656.13094 PMC7041970 31424571

[jfb70560-bib-0076] Wilson, D. S. (1998). Adaptive individual differences within single populations. Philosophical Transactions of the Royal Society B, 353, 199–205.

[jfb70560-bib-0082] Wilson, E. , LaRochelle, L. , Petetta, A. , Landsman, S. J. , & Cooke, S. J. (2026). Air exposure durations influence post‐release swimming activity of muskellunge (*Esox masquinongy*). Aquatic Diversity and Ecology, 100011. 10.1016/j.ade.2026.100011

[jfb70560-bib-0077] Zhang, D. , van der Hoop, J. M. , Petrov, V. , Rocho‐Levine, J. , Moore, M. J. , & Shorter, K. A. (2020). Simulated and experimental estimates of hydrodynamic drag from bio‐logging tags. Marine Mammal Science, 36(1), 136–157.

